# Impact of long‐term high‐altitude residence on cardiopulmonary function in asymptomatic men: A cross‐sectional study

**DOI:** 10.14814/phy2.70864

**Published:** 2026-04-20

**Authors:** Fengjiao Yang, Weixing Tan, Yajun Tian, Qin Wu, Xiaomei Feng, Gangwei Hu, Ou Li

**Affiliations:** ^1^ Air Force Health Care Center for Special Services Hangzhou China

**Keywords:** acclimatization, cardiopulmonary exercise testing, high altitude, hypobaric hypoxia

## Abstract

To evaluate altitude‐stratified differences in static lung function, aerobic capacity, and exercise physiology under standardized normoxic conditions, and identify multiple predictors of peak oxygen uptake (VO_2_) reduction among asymptomatic men after prolonged residence at varying altitudes. We conducted a cross‐sectional study of 103 asymptomatic men stratified by residential altitude: low (<2500 m; *n* = 35), high (2500–3500 m; *n* = 32), and very high (>3500 m; *n* = 36). All underwent spirometry, fasting blood tests, and symptom‐limited cardiopulmonary exercise testing (CPET) in normoxia. Multiple linear regression identified independent predictors of peak VO_2_/kg. Very high‐altitude residents had significantly lower peak VO_2_/kg (−13.4 mL·min^−1^·kg^−1^ vs. low altitude, *p* < 0.001), reduced oxygen pulse, and impaired small‐airway function (MMEF, FEF_75_; *p* < 0.05), despite preserved ventilatory efficiency (VE/VCO_2_ slope, *p* = 0.782). Hemoglobin was elevated at higher altitudes; triglycerides were higher only above 3500 m. Age (*β* = −0.285), regular exercise (≥3 sessions/week; *β* = +3.648), and very high‐altitude residence (*β* = −13.370) independently predicted peak VO_2_/kg (all *p* < 0.001; *R* ^2^ = 0.739). Residence above 3500 m causes persistent cardiopulmonary impairment driven by circulatory limitations and smoking, despite preserved ventilatory efficiency. Normoxic assessment identifies regular exercise (≥3 sessions/week) as a key countermeasure against altitude‐induced deconditioning. Prioritizing smoking cessation and mandatory exercise programs is therefore recommended for long‐term health in high‐altitude personnel.

## 1 INTRODUCTION

High altitude (≥2500 m) is stratified into high (2500–3500 m), very high (3500–5500 m), and extreme (>5500 m) zones (West, 2013), where hypobaric hypoxia poses the primary challenge to cardiopulmonary function, especially for military personnel (Hackett & Roach, 2001; Luks et al., 2023; Muza et al., 2004). Exposure triggers a dose‐dependent decline in maximal oxygen uptake (VO_2_max) of ~6%–10% per 1000 m above 1500 m, driven by reduced arterial oxygen saturation and delivery (Bassett Jr & Howley, 2000; Lundby et al., 2017). Although partial acclimatization occurs over weeks, aerobic capacity rarely returns to sea‐level values even after prolonged residence (Chapman et al., 1998).

Cardiorespiratory fitness (CRF), quantified via cardiopulmonary exercise testing (CPET), integrates pulmonary, cardiovascular, and muscular efficiency to serve as a robust biomarker of functional capacity and health risk (Kodama et al., 2009; Ross et al., 2016). However, in the context of high‐altitude exposure, reliance on static markers such as hemoglobin concentration is often insufficient. Recent evidence underscores that CRF assessed post‐acclimatization provides a superior reflection of true physiological reserve compared to these static measures alone (Willie et al., 2022).

Despite extensive research on acute and subacute altitude exposure, data remain limited regarding the long‐term cardiopulmonary phenotype of individuals following prolonged residence at very high altitude, particularly when assessed under standardized normoxic conditions. Moreover, the extent to which altitude‐induced changes in static lung function translate into impaired ventilatory efficiency during exercise—and whether circulatory or muscular limitations predominate—remains incompletely characterized (Stembridge et al., 2021). Finally, while regular physical activity is known to mitigate deconditioning, evidence‐based exercise prescriptions tailored to high‐altitude recovery populations are lacking (Millet et al., 2020).

In this study, we assess CPET‐derived parameters in asymptomatic males residing across distinct altitudinal zones (low, high, and very high altitude), evaluate their altitude‐adjusted cardiopulmonary performance under normoxic testing conditions, and investigate associations with hematological, metabolic, and lifestyle‐related variables. The findings aim to inform evidence‐based strategies for health surveillance, functional rehabilitation, and targeted interventions among populations recovering in high‐altitude settings.

## 2 SUBJECTS AND METHODS

### 2.1 Participants

From October 2024 to July 2025, asymptomatic male participants were recruited and stratified by residential altitude into three groups. The low‐altitude group (<2500 m; *n* = 35) consisted of individuals primarily from eastern Chinese provinces, including Zhejiang, Jiangsu, Fujian, Guangdong, Shandong, and Anhui, who were long‐term residents at their native altitude. The high‐altitude (2500–3500 m; *n* = 32) and extreme‐high‐altitude (>3500 m; *n* = 36) groups were recruited from Qinghai, Xinjiang, and Tibet; notably, participants in the extreme‐high‐altitude subgroup were specifically stationed in high‐mountain regions of Xinjiang and Tibet.

Prior to testing, all participants underwent a standardized pre‐test preparation protocol to ensure data reliability. Strict pre‐test checks included: (1) abstinence from caffeine, alcohol, and heavy meals for at least 12 h; (2) avoidance of vigorous physical activity for 24 h; (3) no smoking for at least 2 h before the assessment; and (4) suspension of any medications affecting cardiopulmonary function for 24 h, unless medically contraindicated. Upon arrival, a final eligibility check was performed, confirming that participants were free from acute respiratory infections, fever, or excessive fatigue. Resting heart rate, blood pressure, and peripheral oxygen saturation (SpO_2_) were measured to verify hemodynamic stability before proceeding to the cardiopulmonary exercise test.

Regarding exposure duration, the high‐altitude and extreme‐high‐altitude groups had resided at their respective elevations for 24.0 (10.5, 48.0) and 24.0 (12.0, 96.0) months, respectively (Table 1). All high‐altitude participants were confirmed to be acclimatized, with a minimum residence duration of 6 months prior to testing. Written informed consent was obtained from all subjects, who subsequently completed a standardized cardiopulmonary exercise test (CPET).

**TABLE 1:** Baseline characteristics of participants by altitude group.

	Low‐altitude group (*n* = 34)	High‐altitude group (*n* = 32)	Extreme‐high‐altitude group (*n* = 36)	*F/χ* ^2^	*p*
Age (years), M (P25, P75) Height (cm), M (P25, P75)	29.5 (25.0, 35.3)	29.0 (25.3, 33.0)	30.5 (28.3, 35.0)	2.88	0.238
	175.37 ± 4.77	175.78 ± 5.14	172.92 ± 6.17	2.84	0.063
Weight (kg), M (P25, P75)	75.0 (69.8, 80.0)	75.0 (62.1, 79.0)	68.5 (65.0, 79.8)	3.95	0.139
BMI (kg/m^2^), M (P25, P75)	24.8 (22.5, 26.1)	23.9 (20.4, 26.8)	23.8 (22.0, 26.1)	1.71	0.426
Duration at Altitude (months), M (P25, P75)		24.0 (10.5, 48.0)	24.0 (12.0, 96.0)		
Smoking Status, *n* (%)				6.31^b^	0.043
Non‐smokers	26 (76.5%)	19 (59.4%)	17 (47.2%)		
Smokers	8 (23.5%)	13 (40.6%)	19 (52.8%)		
Smoking Index (among smokers), M (P25, P75)	—	100 (30, 150)	120 (45, 210)	1.85^a^	0.396
Exercise Frequency, *n* (%)				1.62	0.445
< 3 times/week	13 (38.2%)	17 (53.1%)	15 (41.7%)		
≥ 3 times/week	21 (61.8%)	15 (46.9%)	21 (58.3%)		

*Note*: Data are mean ± SD, median (P25, P75), or *n* (%). *p* < 0.05 is significant. P25/P75, 25th/75th percentiles. *F*‐value (ANOVA).

Abbreviation: BMI, body mass index.

^a^ Kruskal‐Wallis *H*‐test.

^b^ Chi‐square test. Smoking Index calculated for smokers only.

*Inclusion criteria*: (1) Continuous residence at assigned altitude for ≥6 months; (2) Prior hospitalization >7 days with no clinical symptoms of high‐altitude deacclimatization (e.g., dizziness, somnolence); (3) Achievement of maximal effort during CPET (peak respiratory exchange ratio [RER] >1.1).

*Exclusion criteria*: (1) History of chronic medical conditions (hypertension, coronary artery disease, COPD, chronic bronchitis); (2) Acute illness within 2 weeks (e.g., upper respiratory infection, fever of unknown origin).

The study was approved by the Medical Ethics Committee of the Hangzhou Special Service Recuperation Center of the Air Force (approval no. TLZX20241009‐01).

### 2.2 Procedures

Cardiopulmonary exercise testing (CPET), spirometry, and laboratory assays were conducted in accordance with American Thoracic Society/European Respiratory Society (ATS/ERS) guidelines (Subudhi et al., 2023). For brevity, pre‐test preparation details (e.g., dietary and clothing restrictions) are omitted unless central to methodological novelty.

### 2.3 Statistical analysis

Analyses were performed using SPSS version 22.0 (IBM Corp., Armonk, NY, USA). Continuous variables are presented as mean ± standard deviation (SD) for normally distributed data or median (interquartile range [IQR]) for non‐normal distributions. Group comparisons employed one‐way ANOVA with LSD post hoc tests or Kruskal–Wallis tests with Mann–Whitney *U* pairwise comparisons, as appropriate. Categorical variables were analyzed using chi‐square tests.

Multiple linear regression was used to model peak VO_2_/kg as the dependent variable. Predictor variables included altitude group (reference: low altitude), age, exercise frequency (<3 vs. ≥3 sessions/week), training duration (months), and hemoglobin concentration—all entered simultaneously via the “Enter” method. Statistical significance was defined as *p* < 0.05 (two‐tailed).

## 3 RESULTS

### 3.1 Baseline characteristics across altitude groups

No statistically significant differences were observed among the low‐, high‐, and very high–altitude groups in age, height, body weight, body mass index, or exercise frequency (*p* > 0.05). Notably, the prevalence of current smoking was significantly higher in the very high–altitude group (52.8%) compared to the low‐altitude group (23.5%, *p* < 0.05) (Table 1).

### 3.2 Static pulmonary function

Forced vital capacity (FVC% predicted) and vital capacity (VC% predicted) did not differ significantly across groups (*p* > 0.05). Pairwise comparisons between the high‐ and very high–altitude groups also revealed no significant differences in any static pulmonary parameters (*p* > 0.05).

Compared with the low‐altitude group, both high‐ and very high–altitude groups exhibited significantly lower values for FEV_1_% predicted, peak expiratory flow (PEF), maximal mid‐expiratory flow (MMEF% predicted), FEF_25_% predicted, maximal voluntary ventilation (MVV), and MVV% predicted (*p* < 0.05). Of particular note, the decrease in airflow indices (e.g., PEF, MMEF) was most pronounced in the very high–altitude group, which also harbored the highest proportion of smokers. This disparity raises important considerations regarding lifestyle factors in high‐altitude deployments. Additionally, the high‐altitude group showed significantly reduced FEV_1_/FVC (measured and % predicted) and FEF_50_% predicted relative to the low‐altitude group (*p* < 0.05). The very high–altitude group demonstrated significantly lower FEF_75_% predicted and measured VC compared with the low‐altitude group (*p* < 0.05) (Table 2).

**TABLE 2:** Comparison of static pulmonary function parameters among the three altitude groups.

	Low‐altitude group (*n* = 34)	High‐altitude group (*n* = 32)	Extreme‐high‐altitude group (*n* = 36)	*F/χ* ^2^	*p*
FEV1% predicted	98.85 ± 9.68	91.00 ± 8.07*	92.31 ± 11.58*	6.02	0.003
FVC % predicted	102 (93.75 ± 107.75)	98 (91.25 ± 104)	96 (91.25 ± 106)	2.33	0.311
Measured FEV_1_/FVC	82.39 ± 7.11	77.90 ± 6.80*	79.30 ± 6.64	3.74	0.027
FEV_1_/FVC % predicted	101.00 ± 8.54	95.41 ± 7.83*	97.42 ± 7.84	4.09	0.020
PEF (L/s)	100.35 ± 12.96	90.31 ± 13.22*	93.36 ± 10.12*	6.01	0.003
MMEF % predicted	80 (75.25 ± 110.25)	71 (61.25 ± 82.25)*	77.5 (62.75 ± 87.25)*	12.88	0.002
FEF 75% predicted	77 (59.75 ± 99.5)	65.5 (53.25 ± 79.75)	65 (52.25 ± 75.75)*	7.76	0.021
FEF 50% predicted	93.5 (77.5 ± 120.75)	74.5 (63.5 ± 81.75)*	80 (66.25 ± 92.5)	13.02	0.001
FEF 25% predicted	98.12 ± 16.34	82.06 ± 15.33*	87.22 ± 16.99*	8.46	*p* < 0.001
VC (L)	5.16 ± 0.65	4.99 ± 0.54	4.76 ± 0.58*	4.09	0.02
VC % predicted	100.5 (93 ± 107.75)	97 (88.25 ± 101)	92.5 (88 ± 102.5)	3.78	0.151
MVV (L/min)	161.85 (147.1 ± 180.2)	140.3 (132.15 ± 150.125)*	140.55 (129.78 ± 158.7)*	22.52	*p* < 0.001
MVV % predicted	114.00 ± 14.14	98.34 ± 12.05^a^	102.11 ± 12.80^a^	13.15	*p* < 0.001

*Note*: Data are presented as mean ± SD or median (P25, P75). Comparisons used one‐way ANOVA or Kruskal–Wallis test with appropriate post‐hoc corrections.

Abbreviations: FEF, forced expiratory flow at specified percentages of FVC; FEV_1_, forced expiratory volume in 1 s; FVC, forced vital capacity; MMEF, maximal mid‐expiratory flow; MVV, maximum voluntary ventilation; PEF, peak expiratory flow; VC, vital capacity.

* *p* < 0.05 vs. Low‐Altitude Group.

### 3.3 Cardiopulmonary exercise responses

#### 3.3.1 Aerobic Capacity

Peak VO_2_/kg, peak VO_2_% predicted, oxygen uptake efficiency slope (OUES), metabolic equivalents (METs), and peak power output were all significantly lower in the very high–altitude group than in the low‐altitude group (*p* < 0.05). No significant differences were observed between the high‐ and very high–altitude groups. Oxygen uptake at the anaerobic threshold (VO_2_@AT) did not differ across groups (*p* = 0.111).

#### 3.3.2 Circulatory Function

Peak oxygen pulse (peak VO_2_/HR) followed a graded pattern: lowest in the very high–altitude group, intermediate in the high‐altitude group, and highest in the low‐altitude group (*p* < 0.05). In contrast, the oxygen uptake‐to‐work rate slope (∆VO_2_/∆W) was comparable across all three groups (*p* = 0.162).

#### 3.3.3 Ventilatory and Gas Exchange Parameters

While the VE/VCO_2_ slope, breathing reserve (BR), and respiratory frequency (Bf) did not differ significantly among groups (*p* > 0.05), ventilatory efficiency relative to oxygen uptake showed altitude‐dependent changes. Specifically, the VE/VO_2_@AT was significantly elevated in the very high–altitude group compared to the low‐altitude group (Low: 24.2 (22.93, 25.83) vs. Very High: 26.05 (24.2, 27.8), *p* < 0.05), reflecting the heightened hypoxic ventilatory drive required to maintain oxygenation despite increased dead space ventilation.

#### 3.3.4 Cardiac Autonomic Regulation

Heart rate reserve, 1‐min heart rate recovery (HRR), resting diastolic blood pressure, and peak diastolic blood pressure were similar across groups (*p* > 0.05). However, a distinct dissociation in systolic blood pressure responses was observed: peak systolic blood pressure was significantly lower in the very high–altitude group than in the low‐altitude group (*p* < 0.05), while resting systolic blood pressure was significantly higher in the high‐altitude group compared with the low‐altitude group (*p* < 0.05) (Table 3).

**TABLE 3:** Comparison of cardiopulmonary function parameters during exercise among the three altitude groups.

	Low‐altitude group (*n* = 34)	High‐altitude group (*n* = 32)	Extreme‐high‐altitude group (*n* = 36)	*F/χ* ^2^	*p*
Peak Power Output (W)	208.62 ± 36.14	192.53 ± 41.97	175.39 ± 30.17*	7.38	0.001
Peak VO_2_/kg (mL·min^−1^·kg^−1^)	32.52 ± 5.21	31.80 ± 5.32	29.42 ± 4.67*	3.61	0.031
Peak VO_2_% Predicted	81.88 ± 11.81	78.16 ± 12.79	73.83 ± 10.36*	4.19	0.018
VO_2_@AT (mL·min^−1^·kg^−1^)	20.4 (16.6, 23.4)	20 (16.53, 24.9)	18.8 (16.48, 20.5)	4.40	0.111
METs	9.29 ± 1.47	9.09 ± 1.52	8.42 ± 1.33*	3.52	0.033
Breathing Reserve (%)	46.39 ± 13.27	43.27 ± 15.67	44.12 ± 17.05	0.37	0.695
Respiratory Rate (breaths/min)	39.00 ± 8.61	37.38 ± 8.87	39.06 ± 9.04	0.38	0.683
Peak Oxygen Pulse (mL/beat)	14.43 ± 2.27	14.08 ± 2.63	12.95 ± 2.06*,**	3.90	0.023
∆VO_2_/∆W (mL·min^−1^·W^−1^)	9.52 (9.13, 10.12)	9.46 (9.07, 10.08)	9.24 (8.63, 9.69)	3.64	0.162
Resting Systolic Blood Pressure (mmHg)	104 (99, 108)	110 (104, 119)*	106.5 (101.25, 115)	7.51	0.023
Resting Diastolic Blood Pressure (mmHg)	70 (66.75, 77.5)	73 (68, 81)	72 (68, 77.57)	1.341	0.511
Peak Systolic Blood Pressure (mmHg)	188.5 (172.5, 206.25)	176 (162, 192)	164.5 (155.75, 181)*	12.14	0.002
Peak Diastolic Blood Pressure (mmHg)	85.71 ± 13.33	84.72 ± 14.50	83.11 ± 12.79	0.30	0.743
Respiratory Quotient (RQ)	1.23 (1.16, 1.28)	1.225 (1.16, 1.28)	1.25 (1.19, 1.31)	2.24	0.326
VE/VO2@AT	24.2 (22.93, 25.83)	25.3 (23.63, 27.93)	26.05 (24.2, 27.8)*	7.54	0.023
VE/VCO_2_ Slope	22.95 (21.9, 24.3)	22.85 (20.73, 26.08)	24.1 (22.15, 25.9)	2.36	0.308
Oxygen Uptake Efficiency Slope (OUES)	2606 (2337.75, 2845.75)	2392.5 (2050.5, 2979.5)	2276 (2093.25, 2516.5)*	9.52	0.009
Minute Ventilation (VE, L/min)	88 (72.95, 99.38)	73.45 (67.25, 101.83)	77.4 (64.33, 95.73)	2.85	0.241
Peak Heart Rate (beats/min)	171.5 (161, 179.25)	167 (152.5, 173)	162.5 (147, 173)	4.61	0.100
Heart Rate Reserve (beats/min)	18 (11.75, 27)	24 (15.5, 34.75)	23.5 (14, 33.75)	4.20	0.122
1‐Minute Heart Rate Recovery (beats/min)	23 (19, 28.25)	27 (21.25, 31)	24 (21.25, 29.75)	4.08	0.130

*Note*: Data are mean ± SD or median (P25, P75). Pairwise comparisons were adjusted using the Bonferroni correction. @AT denotes values at anaerobic threshold.

Abbreviations: AT, anaerobic threshold; HRR, heart rate recovery; METs, metabolic equivalents; OUES, oxygen uptake efficiency slope; RQ, respiratory quotient; SBP/DBP, systolic/diastolic blood pressure; VE, minute ventilation; VO_2_, oxygen uptake.

* *p* < 0.05 vs. Low‐Altitude Group.

** *p* < 0.05 vs. High‐Altitude Group.

### 3.4 Laboratory findings

Fasting blood glucose and total cholesterol levels were comparable across groups (*p* > 0.05). Hemoglobin concentration and triglyceride levels did not differ between the high‐ and very high–altitude groups (*p* > 0.05). Hemoglobin was significantly elevated in both high‐ and very high–altitude groups relative to the low‐altitude group (*p* < 0.05). Triglyceride levels were also significantly higher in the very high–altitude group than in the low‐altitude group (*p* < 0.05) (Table 4).

**TABLE 4:** Comparison of laboratory parameters among the three altitude groups.

	Low‐altitude group (*n* = 34)	High‐altitude group (*n* = 32)	Extreme‐high‐altitude group (*n* = 36)	*F/χ* ^2^	*p*
Hemoglobin (g/L)	157.5 (150.75, 168)	167.59 (157, 179)*	164 (156.25, 186.25)*	9.25	0.010
Fasting Blood Glucose (mmol/L)	5.03 ± 0.39	4.95 ± 0.42	5.07 ± 0.52	0.69	0.506
Total Cholesterol (mmol/L)	4.58 ± 1.03	4.43 ± 0.90	4.75 ± 0.97	0.95	0.389
Triglycerides (mmol/L)	1.01 (0.71, 1.34)	1.07 (0.82, 2.03)	1.33 (0.89, 1.83)*	6.43	0.040

*Note*: Data are mean ± SD or median (P25, P75). Statistical significance determined by ANOVA or Kruskal–Wallis test.

Abbreviations: FBG, fasting blood glucose; Hb, hemoglobin; TC, total cholesterol; TG, triglycerides.

* *p* < 0.05 vs. Low‐Altitude Group.

### 3.5 Multiple linear regression analysis

Variables with *p* < 0.1 in univariate analyses—namely age, height, exercise frequency (dichotomized as <3 vs. ≥3 sessions/week), altitude group (categorized using dummy variables with the low‐altitude group as reference), training duration (months), and hemoglobin concentration—were entered simultaneously into a multiple linear regression model using the enter method.

The final model identified three independent predictors of peak VO_2_ (mL·min^−1^·kg^−1^):

- Each additional year of age was associated with a 0.285 mL·min^−1^·kg^−1^ decrease (*p* < 0.001);
- Regular exercisers (≥3 sessions/week) had a 3.648 mL·min^−1^·kg^−1^ higher peak VO_2_ than less active participants (*p* < 0.001);
- Residence at very high altitude was associated with a 13.370 mL·min^−1^·kg^−1^ reduction compared with low altitude (*p* < 0.001).

The resulting regression equation is:

Peak VO_2_ = 40.928–0.285 × age−13.370 × very high altitude + 3.648 × exercise frequency.

Practically, this model suggests that maintaining a regular exercise routine (≥3 sessions/week) can partially offset the aerobic capacity loss associated with very high‐altitude residence, improving peak VO_2_ by approximately 3.6 mL·min^−1^·kg^−1^.

**FIGURE 1:**
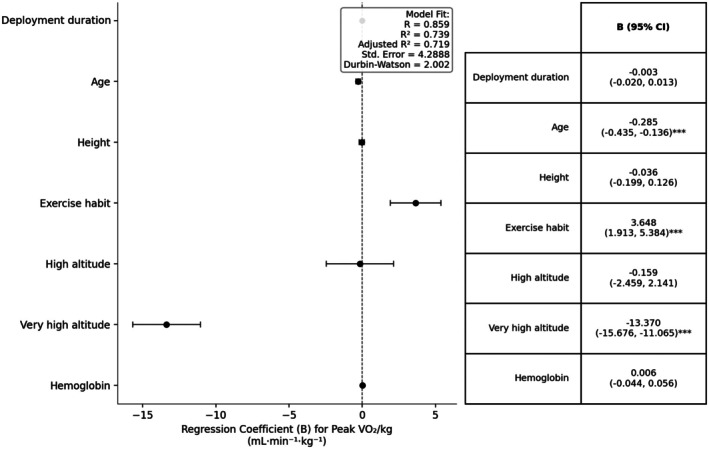
Forest plot of the multivariable linear regression model assessing the association between seven predictors and peak oxygen uptake (Peak VO_2_/kg) in adult males. Each dot represents the unstandardized regression coefficient (B), with horizontal lines indicating the 95% confidence interval (CI). Statistical significance is denoted by asterisks: *p < 0.05, **p < 0.01, ***p < 0.001; non‐significant associations are shown without symbols. The accompanying table (right panel) summarizes the B estimates, 95% CIs, and significance markers for each variable. Model fit statistics are displayed in the upper‐right inset: *R* = 0.859, *R* ^2^ = 0.739, Adjusted *R* ^2^ = 0.719, Standard Error of the Estimate = 4.2888, and Durbin–Watson statistic = 2.002.

The model explained 73.9% of the variance in peak VO_2_ (*R* ^2^ = 0.739). All variance inflation factors (VIFs) were <2, indicating negligible multicollinearity, and the Durbin–Watson statistic (2.002) suggested no residual autocorrelation (Figure 1).

## 4 DISCUSSION

Crucially, all CPETs were conducted at sea level (plain regions). High‐altitude recruits were tested within 1 week of their arrival at the low‐altitude facility; this specific timing window allowed us to eliminate acute hypoxic confounding factors associated with immediate ascent while ensuring that the long‐term physiological imprint of chronic exposure had not yet been reversed by re‐oxygenation. This approach effectively isolates the persistent physiological adaptations resulting from chronic residence, representing a methodological strength aligned with recent recommendations for post‐acclimatization phenotyping (Subudhi et al., 2023).

### 4.1 Pulmonary function: Resting impairment without exercise limitation

We observed reduced FEV_1_, PEF, and small‐airway flow rates (MMEF, FEF_25–75_) in high‐ and very high–altitude residents compared with lowlanders. These findings suggest that chronic hypoxia may induce subtle obstructive changes, possibly through hypoxia‐mediated airway inflammation, increased mucus viscosity, or smooth muscle hyperreactivity (Ge et al., 2019). Notably, unlike some prior reports showing preserved or enhanced expiratory flows in lifelong high‐altitude natives (Brutsaert et al., 2018), our cohort—comprising non‐native, asymptomatic military personnel—exhibited uniformly lower values. This discrepancy likely reflects differences in developmental acclimatization (lifelong vs. acquired exposure). Furthermore, the pronounced airflow limitation in the very high‐altitude group appears to be exacerbated by a significantly higher prevalence of smoking (52.8% vs. 23.5%). This elevated rate is likely attributable to the unique psychosocial stressors of remote high‐mountain deployments, where smoking serves as a prevalent coping mechanism for isolation and harsh climatic conditions. Consequently, the observed pulmonary deficits may represent a synergistic interaction between chronic hypoxic stress and tobacco‐induced airway injury, rather than hypoxia alone—a ‘double‐hit’ phenomenon that underscores the vulnerability of acquired high‐altitude residents to environmental and behavioral risk factors (Zhou et al., 2020).

Critically, despite these resting deficits, ventilatory efficiency during exercise (VE/VO_2_@AT, VE/VCO_2_ slope, breathing reserve) was preserved across all groups. This indicates that the lungs retain sufficient functional reserve to meet metabolic demands during exertion, consistent with the concept that exercise limitation at altitude is predominantly circulatory or muscular rather than ventilatory (Dempsey et al., 2020). The primary reason for test termination—lower limb fatigue—further supports this interpretation.

### 4.2 Cardiopulmonary endurance: A threshold effect at very high altitude

Peak VO_2_/kg and oxygen pulse (a surrogate for stroke volume (Wasserman et al., 2019)) declined progressively with altitude, with significant impairment evident only in the very high–altitude group. This non‐linear response suggests a physiological threshold around 3500–4000 m, beyond which compensatory mechanisms (e.g., erythropoiesis, capillary density) become insufficient to maintain aerobic capacity—a phenomenon recently termed the “altitude ceiling effect” (Siebenmann et al., 2021). Our finding aligns with longitudinal data showing VO_2_max plateaus or declines despite continued erythropoietic stimulation above 4000 m (Gore et al., 2019).

The lack of difference between high‐ and very high–altitude groups in some parameters may reflect limited statistical power or interindividual variability in acclimatization. Nevertheless, the 13.4 mL·min^−1^·kg^−1^ deficit in peak VO_2_ among very high–altitude residents is clinically meaningful—equivalent to ~30% of average young adult VO_2_max—and exceeds thresholds linked to reduced functional independence (Myers et al., 2020).

### 4.3 Metabolic and autonomic adaptations

Elevated hemoglobin in both high‐altitude groups confirms effective erythropoietic adaptation to chronic hypoxia (Gassmann et al., 2022). However, the isolated increase in triglycerides at very high altitude suggests altitude may disrupt lipid metabolism, possibly via sympathetic overactivation or altered hepatic lipase activity (Zhang et al., 2021)—though dietary confounders cannot be excluded.

While the elevated resting systolic blood pressure in the high‐altitude group diverges from classical reports of hypotension in acclimatized residents—potentially reflecting incomplete adaptation or unmasked sympathetic tone under normoxic testing (Gerile, 2018)—the significantly attenuated peak systolic response during exercise (Extreme‐High: 164.5 vs. Low: 188.5 mmHg; *p* = 0.002) represents a definitive physiological adaptation rather than cardiac impairment. This blunted pressor capacity is primarily driven by three synergistic mechanisms: (1) a constrained sympathetic reserve secondary to chronically elevated basal tone, which limits further neural recruitment during exertion (Lewis et al., 2022; Xie et al., 2021); (2) mechanical restrictions on stroke volume, corroborated by reduced peak oxygen pulse (Lawley et al., 2021; Stembridge et al., 2020); and (3) adaptive peripheral vasodilation that prioritizes tissue perfusion efficiency over pressure generation (Januszkiewicz et al., 2023). Collectively, these factors shift the high‐altitude hemodynamic profile from ‘pressure‐driven’ to ‘efficiency‐driven’.

### 4.4 Exercise as a modifiable protective factor

Our regression model identifies regular exercise (≥3 sessions/week) as a strong positive predictor of peak VO_2_, independent of altitude. This reinforces recent calls for structured physical activity programs in high‐altitude military settings (Xu et al., 2022). While WHO guidelines recommend 150–300 min/week of moderate‐intensity activity (World Health Organization, 2020), our data support a minimum frequency of three sessions per week as a practical target for preserving cardiorespiratory fitness during high‐altitude deployment or recovery.

## 5 CONCLUSION

This study, utilizing a unique post‐acclimatization phenotyping protocol conducted at sea level, demonstrates that chronic high‐altitude residence induces persistent, altitude‐dependent physiological adaptations among asymptomatic men. While resting pulmonary function shows subtle obstructive impairments—exacerbated by a synergistic “double‐hit” of chronic hypoxia and high smoking prevalence in extreme‐altitude groups—ventilatory efficiency during exercise remains preserved, indicating that exertion limits are primarily circulatory rather than ventilatory. Crucially, we identified a physiological threshold around 3500 m, beyond which aerobic capacity (VO_2_maxVO_2_max) and stroke volume decline significantly, marking an “altitude ceiling” where compensatory mechanisms become insufficient. Despite these challenges, regular exercise (≥3 sessions/week) emerged as a potent, modifiable protective factor capable of partially offsetting aerobic deficits. These findings underscore the necessity for targeted health interventions, specifically smoking cessation and structured exercise programs, to maintain operational readiness and long‐term cardiopulmonary health in personnel deployed to extreme high‐altitude environments.

## AUTHOR CONTRIBUTIONS

All authors made substantial contributions to the conception, design, acquisition, analysis, or interpretation of the data. Specifically: Fengjiao Yang and Ou Li conceived the study and designed the protocol. Fengjiao Yang, Weixing Tan, and Qin Wu performed the data collection and cardiopulmonary exercise testing. Fengjiao Yang and Weixing Tan contributed to the literature review, critical revision of the manuscript for important intellectual content, and final proofreading. Weixing Tan and Xiaomei Feng (New Author) contributed to the re‐analysis of gas exchange data, refined the statistical modeling, and drafted the revised Discussion section. Yajun Tian and Gangwei Hu supervised the project, interpreted the results, and finalized the manuscript. All authors have read and agreed to the published version of the manuscript. The order of authors was determined by mutual agreement based on the magnitude of each individual's contribution.

## FUNDING INFORMATION

This research received no specific grant from any funding agency in the public, commercial, or not‐for‐profit sectors.

## CONFLICT OF INTEREST STATEMENT

The authors declare no conflicts of interest, financial or otherwise, related to the content of this article.

## ETHICS STATEMENT

The study protocol was reviewed and approved by the Medical Ethics Committee of the Hangzhou Special Service Recuperation Center of the Air Force (Approval No. TLZX20241009‐01). Written informed consent was obtained from all participants prior to their inclusion in the study. The study was conducted in accordance with the principles of the Declaration of Helsinki. All authors have reviewed and approved the final version of the manuscript and agree to be accountable for all aspects of the work.

## Data Availability

The data that support the findings of this study are available from the corresponding author upon reasonable request. Due to the nature of the data involving military personnel, access may be subject to confidentiality agreements and institutional approval.
